# Short Report: Circulating microRNAs are associated with incident diabetes over 10 years in Japanese Americans

**DOI:** 10.1038/s41598-020-63606-3

**Published:** 2020-04-16

**Authors:** Pandora L. Wander, Daniel A. Enquobahrie, Theo K. Bammler, Sengkeo Srinouanprachanh, James MacDonald, Steven E. Kahn, Donna Leonetti, Wilfred Y. Fujimoto, Edward J. Boyko

**Affiliations:** 1 Department of Medicine,University of Washington, Seattle, WA United States; 2 Veterans Affairs Puget Sound Health Care System, Seattle, WA United States; 3 Department of Epidemiology,University of Washington, Seattle, WA United States; 4 Department of Environmental and Occupational Health Sciences,University of Washington, Seattle, WA United States; 5 Department of Anthropology, University of Washington, Seattle, WA United States

**Keywords:** Pre-diabetes, Molecular medicine

## Abstract

Epigenetic changes precede the development of diabetes by many years, providing clues to its pathogenesis. We explored whether the epigenetic markers, circulating microRNAs (miRNAs), were associated with incident diabetes in Japanese Americans. We conducted a pilot study (n = 10) using plasma from age- and sex-matched participants who did or did not develop diabetes in the Japanese American Community Diabetes Study, an observational study of diabetes risk factors. Extraction and high-throughput sequencing of miRNAs were performed using samples collected at baseline. Regression models were fit comparing circulating miRNAs (N = 1640) among individuals who did or did not develop incident diabetes at 10-year follow-up. Participants averaged 51.7 years of age at baseline; 60% were male. We identified 36 miRNAs present at different (10 higher and 26 lower) levels in individuals who developed diabetes compared to those who did not (log_2_fold change ≥1.25 and false discovery rate ≤5%). These included miRNAs with functions in skeletal muscle insulin metabolism (miR-106b and miR-20b-5p) and miRNAs with functions in both skeletal muscle insulin metabolism and cell cycle regulation in endocrine pancreas (miR-15a and miR-17). Circulating miRNAs were associated with subsequent development of diabetes among Japanese Americans over 10 years of follow-up. Results are preliminary. Large-scale miRNA sequencing studies could inform our understanding of diabetes pathogenesis and development of therapies, based on gene expression regulation, that target diabetes.

## Introduction

A better understanding of tissue level changes in gene expression and regulation occurring early in the course of type 2 diabetes could provide information about its pathogenesis and inform the development of predictive biomarkers. Unfortunately, tissue-specific biomarkers are rarely measured in longitudinal observational studies because tissue-collection protocols are invasive, expensive and time consuming, limiting our understanding of tissue-level abnormalities that precede diabetes. Circulating microRNAs (miRNAs) are short non-coding RNAs that regulate tissue gene expression^1^. They are actively secreted into the circulation^2^, where they are markers of tissue regulation of gene expression as well as mediators of inter-organ cross-talk^3^. Using a real-time polymerase chain reaction (RT-PCR), approach two studies have identified altered patterns in circulating miRNAs that precede the development of diabetes by up to 15 years in Europeans^4,5^. To our knowledge, no prior study has been conducted in Japanese Americans, a group at high risk of type 2 diabetes. In addition, prior studies involved candidate miRNA profiling, limiting potential discovery that follows comprehensive miRNA profiling. Thus, we used next-generation RNA sequencing to examine whether circulating miRNAs preceded the development of diabetes in Japanese Americans after 10 years of follow-up.

## Methods

### Study setting and study population

Study participants were selected from the Japanese American Community Diabetes Study (JACDS), a community-based cohort of individuals of 100% Japanese ancestry. Selection and recruitment procedures have been described previously^6^. For this analysis, we randomly selected five individuals from among those without prevalent diabetes at baseline and incident diabetes at the year 10 follow-up visit and five controls individually matched by age and sex who remained free from diabetes for 10 years. The study was approved by the University of Washington Human Subjects Division. Participants provided written informed consent, and all research was performed in accordance with relevant guidelines and regulations.

### Data collection

Data and sample collection were done at the General Clinical Research Center at the University of Washington, Seattle, by trained staff. Blood specimens were drawn at enrollment and at a follow-up visit at 10–11 years after at least a 10-hour fast. Plasma was isolated and stored at −80 °C. Presence of diabetes was assessed at baseline and follow-up using a two-hour oral glucose tolerance test (75 gram load) and defined as fasting glucose ≥126 mg/dl (7.0 m mol/L) and/or 2-h glucose ≥200 mg/dl (11.1 mmol/L)^7^, or use of diabetes medication.

### Pre-processing, extraction and profiling of circulating miRNAs

Thawed samples were spun at ~2500 RPMs for five minutes to completely clear plasma of cells. Small RNAs were extracted from 500 μL plasma aliquots using the Exiqon (now Qiagen) miRCURY^TM^ RNA Biofluids Isolation Kit (Exiqon, Woburn, MA). Integrity, purity and quantity of purified miRNA was assessed using spectrophotometry and an Agilent 2100 Bioanalyzer capillary electrophoresis system (Agilent Technologies Inc, Palo Alto, CA). The Qiagen QIAseq miRNA NGS Library Kit was used for library preparation. MiRNAs were sequenced using an Illumina sequencer. Lab personnel were blinded to participant outcomes. Additional technical details are provided in Supplemental Materials.

### Statistical and bioinformatics analyses

Number (%) and mean (standard deviation) describe study population characteristics. Analyses were conducted in R version 3.4.0. Samples were normalized using a weighted trimmed mean of M-values (TMM), which calculates a normalization factor that is used to scale the library sizes. Because count data are not normally distributed and may have transcripts with zero counts^8^, a linear model based on the negative binomial distribution in the Bioconductor edgeR package^9^ using quasi-likelihood F-tests^10^ was used. Exploratory principal component analysis showed an apparent large batch effect captured by the first principal component; therefore, the first principal component was included as an adjustment variable, along with two surrogate variables detected using the Bioconductor sva package^11^. To protect against choosing miRNA transcripts that may be differentially expressed at a statistically significant but low-fold–change level that is not biologically meaningful, miRNAs with at least a 25% difference^12^ in expression as well as a <5% false discovery rate (FDR) were identified as significantly different, as has been previously reported^13^. We used the Bioconductor sizepower package to estimate study power, *post hoc*. The average standard error was 0.999, and we tested for a 1.25-log2fold difference. We found 36 out of 1640 miRNAs significant. Accepting 10 false positives, we calculate we had 31% power to identify differences in circulating miRNAs between the groups. The Core Analysis feature of the Ingenuity Pathway Analysis (IPA) software program (Built version–486617 M; Content version–33559992; Ingenuity Systems, A Qiagen Company, Redwood City, CA) was used to identify transcriptional networks using microRNAs that were present at different levels in cases than in controls^14^. IPA’s microRNA Target Filter to identify mRNA targets was also used, restricting the search to “experimentally validated” targets. IPA Core Analysis was performed on the lists of targets that were identified as described above by IPA’s microRNA Target Filter feature.

### Ethics approval and consent to participate

This study was approved by the institutional review board at the University of Washington as # STUDY00001823, and all participants provided written informed consent.

## Results

**Figure 1:**
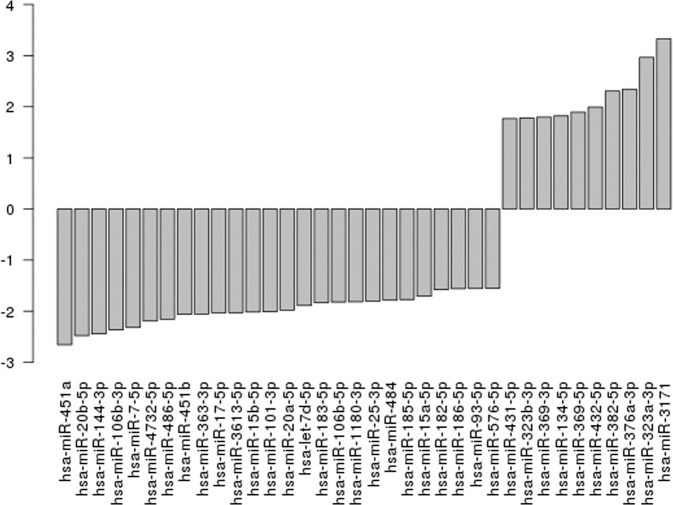
Barplot of log fold changes for each miRNA. For aid in interpretation we have sorted the miRNA transcripts by fold change.

Baseline characteristics of the cohort are shown in Table 1. After excluding miRNA transcripts with mean log counts per million (CPM) ≤ 2.5, 1640 transcripts were carried forward. Table 2 and Fig. 1 show log_2_fold-change differences (logFC), log counts per million (logCPM), and false discovery rates (FDR) for circulating miRNAs measured at baseline that differ between individuals with and without incident diabetes after 10 years of follow-up (n = 36; FDR < 0.05, ≥1.25-fold change). Identified miRNAs included miR-20b-5p (logFC −2.48, FDR 0.0042), miR-363-3p (logFC −2.06, FDR 0.0042), miR-7-5p (logFC −2.32, FDR 0.0042), miR-144-3p (logFC −2.44, FDR 0.0074), miR-20a (logFC −1.98, FDR 0.0074), miR-451b (logFC −2.06, 0.0074), miR-15b-5p (logFC −2.02, FDR 0.0109), and miR-15a-5p (logFC −1.71, FDR 0.0156), which were all lower in individuals who developed diabetes compared to those who did not. A pathway analysis suggested identified miRNAs may play a role in cell-cycle regulation (Supplementary Table 1, Supplementary Fig. 1). The top network represented by experimentally validated gene (mRNA) targets of microRNAs was “Cell cycle, embryonic development.”

**Table 1:** Baseline demographic and laboratory values for Japanese American Community Diabetes Study (JACDS) participants without diabetes at baseline and participants chosen for this miRNA subcohort study, stratified by the presence of incident diabetes at 10-year follow-up.

	JACDS participants without diabetes at baseline		miRNA subcohort*	
	Incident diabetes	No diabetes	Incident diabetes	No diabetes
	n = 103	n = 415	n = 5	n = 5
Mean age, years (SD)	57.9 (10.9)	50.7 (11.9)	51.6 (13.8)	51.8 (13.8)
Sex, % male (n)	54 (56)	50 (208)	60 (3)	60 (3)
Mean body-mass index, kg/m^2^ (SD)	25.3 (3.6)	23.8 (3.1)	25.0 (3.5)	23.6 (3.3)
Mean waist circumference, cm (SD)	89.3 (8.0)	85.0 (8.6)	85.0 (11.9)	79.4 (12.2)
Mean baseline fasting glucose, mmol/L (SD)	5.6 (0.5)	5.1 (0.6)	5.4 (0.5)	5.5 (0.7)
Mean baseline 2-hour glucose from OGTT, mmol/L (SD)	8.8 (1.3)	6.9 (1.6)	8.5 (1.4)	7.7 (2.0)
Presence of impaired fasting glucose, % yes (n)	53 (55)	20 (85)	40 (2)	20 (1)
Presence of impaired glucose tolerance, % yes (n)	84 (86)	30 (125)	80 (4)	60 (3)
Family history of diabetes, % yes (n)	55 (57)	33 (137)	60 (3)	60 (3)
Smoker, % current (n)	16 (16)	12 (51)	0 (0)	40 (2)

*Cases and controls matched by sex and age (within two years).

**Table 2:** Log_2_ fold-change differences (logFC), mean log_2_ counts per million (log counts per million; CPM), and false discovery rates (FDR) for circulating miRNAs that differ between individuals with and without incident diabetes after 10 years of follow-up.

Symbol	logFC	logCPM	FDR
hsa-miR-363-3p	−2.06	9.24	0.0042
hsa-miR-7-5p	−2.32	7.97	0.0042
hsa-miR-144-3p	−2.44	10.50	0.0074
hsa-miR-20a-5p	−1.98	9.37	0.0074
hsa-miR-451b	−2.06	7.43	0.0074
hsa-miR-106b-3p	−2.36	7.72	0.0078
hsa-miR-451a	−2.66	14.60	0.0087
hsa-miR-15b-5p	−2.02	11.30	0.0109
hsa-miR-183-5p	−1.83	9.01	0.0109
hsa-miR-484	−1.79	9.80	0.0109
hsa-miR-382-5p	2.31	10.60	0.0113
hsa-miR-323a-3p	2.96	6.30	0.0134
hsa-miR-15a-5p	−1.71	10.70	0.0156
hsa-miR-17-5p	−2.03	8.41	0.0156
hsa-miR-25-3p	−1.80	12.40	0.0185
hsa-miR-134-5p	1.82	9.00	0.0206
hsa-let-7d-5p	−1.89	11.80	0.0260
hsa-miR-106b-5p	−1.82	7.64	0.0260
hsa-miR-182-5p	−1.58	9.52	0.0260
hsa-miR-323b-3p	1.78	6.68	0.0260
hsa-miR-369-5p	1.89	6.66	0.0260
hsa-miR-376a-3p	2.34	5.64	0.0260
hsa-miR-432-5p	1.99	9.78	0.0260
hsa-miR-4732-5p	−2.19	8.64	0.0260
hsa-miR-93-5p	−1.56	11.80	0.0260
hsa-miR-486-5p	−2.16	15.90	0.0270
hsa-miR-101-3p	−2.01	14.20	0.0315
hsa-miR-1180-3p	−1.82	7.34	0.0315
hsa-miR-185-5p	−1.77	11.60	0.0315
hsa-miR-186-5p	−1.56	8.83	0.0315
hsa-miR-3613-5p	−2.03	9.31	0.0315
hsa-miR-576-5p	−1.55	7.09	0.0315
hsa-miR-3171	3.33	4.81	0.0375
hsa-miR-431-5p	1.77	7.99	0.0375
hsa-miR-369-3p	1.79	7.20	0.0408

Significance was set at an FDR < 0.05 and a|logFC | ≥1.25.

## Discussion

In this exploratory analysis using archival samples from a community-based study of diabetes risk factors in Japanese Americans, we identified 36 miRNAs (FDR < 0.05, >1.25-fold change) present at different levels in plasma of individuals who did versus did not develop diabetes up to 10 years later. Although the sample size is small, this report establishes new findings and extends previous findings in this area in several important ways. First, we extended the study of circulating miRNAs with long-term follow-up for incident diabetes to a new population, Asian-Americans. Second, by using a comprehensive sequencing approach, we identified circulating miR-106b, miR-17 and miR-20b-5p not reported in previous long-term longitudinal studies. These miRNAs have putative roles in the pathogenesis of diabetes via actions in skeletal muscle and/or pancreatic islet cells. Lastly, we replicated previous observations^4,5^ that lower baseline levels of miR-15a were associated with incident diabetes, suggestingits potential as a predictive diabetes biomarker.

In the Spanish CORDIOPREV cohort (n = 462), higher levels of miR-150 and miR-30a-5p and lower levels of miR-15a and miR-375 were associated with higher diabetes risk at 60 months^4^. In the Bruneck (Italy) cohort, individuals with incident diabetes at 10 years (n = 19) had lower baseline plasma levels of miR-15a, miR-29b, miR-126 and miR-223 and higher levels of miR-28-3p than matched controls^5^. In our sample, except for miR-15a, the other associations were not replicated. A secondary analysis of the Practicing Restorative Yoga vs. Stretching for the Metabolic Syndrome study examined associations of circulating miRNAs with incident diabetes over 12 months of follow-up^15^. As in our study, baseline levels of miR-106b, miR-20b, miR-363, miR-486, miR-532 and miR-93 were associated with incident diabetes, as well as with response to a lifestyle intervention^15^.

In skeletal muscle from monozygotic twins discordant for type 2 diabetes, expression of miR-106b, miR-15b, miR-17, miR-20a, miR-20b, miR-25, miR-363, miR-451, miR-93 (identified in the current study), and others were downregulated in twins with type 2 diabetes compared to twins without diabetes^16^. In a community-based sample, miR-106b, miR-144, miR-15b, miR-451, and miR-93 were upregulated and miR-15a downregulated in skeletal muscle of individuals with type 2 diabetes compared to individuals with normal glucose tolerance^17^. In another study, higher levels of *both* skeletal muscle miR-15a and miR-15b were associated with higher fasting and 2-hour glucose in adults with and without diabetes^18^. Inconsistency of identified miRNAs in previous as well as the current study could be due to racial and ethnic differences in the study populations, heterogeneous mechanisms contributing to diabetes pathogenesis across the populations studied,or differences in methods (sample source, processing, and profiling). Carefully designed studies incorporating repeated measurement of miRNAs over time in cohorts at high diabetes risk will be needed to clarify these relationships.

Identified miRNAs including miR-17, miR-106b and miR-20b-5p have functions in skeletal muscle insulin metabolism. In skeletal muscle, miR-17^19^ and miR-106b^20^ downregulate components of the glucose transporter 4 pathway, while overexpression of miR-20b-5p impairs insulin signaling and suppresses genes in pathways related to immune function (AKTIP and STAT3)^21^. Some identified miRNAs have functions in islet cells as well. In pancreatic cell lines, miR-17 downregulates thioredoxin-interacting protein, a pro-apoptotic regulator of beta-cells in diabetes^22^. In mouse islets, miR-7 (miR-7a) targets multiple components of the mTOR signaling pathway, and its inhibition promotes adult beta-cell replication^23^. In rats, miR-25 negatively regulates expression of insulin I messenger transcripts^24^. Last, islet cell expression of miR-15a is upregulated in the presence of brief (1 h) exposure to hyperglycemia but depressed after a longer period of exposure^25^. At the same time, overexpression of miR-15a increases insulin levels and its repression inhibits insulin synthesis by inhibiting uncoupling protein-2 (UCP-2) gene expression^25^. Five miRNAs identified in this study are members of the highly conserved polycistronic miR-17~92 family (miR-17, miR-20, miR-25, miR-93, and miR-106b)^26^, which may be important in cell-cycle regulation, as suggested by our bioinformatics analysis results.

An important limitation of our pilot study is that it was underpowered to detect an exhaustive list of circulating miRNAs associated with incident diabetes. We used conservative correction for multiple testing to mitigate risk of type II error. Results are preliminary and need to be replicated in other populations. In addition, at baseline the groups differed in distribution of impaired fasting glucose (IFG) and impaired glucose tolerance (IGT). To further characterize baseline glycemic status, we used 30-, 60-, 120-, and 180-minute values of glucose and insulin from baseline oral glucose tolerance tests to calculate Matsuda and insulinogenic indices (IGI) for study participants. These values were very similar between the groups (Matsuda 3.1 ± 0.7 vs. 3.2 ± 1.3, p = 0.879; IGI 0.8 ± 1.0 vs. 0.7 ± 0.4, p = 0.883), suggesting that differences in baseline miRNA levels were unlikely to be attributable to differences in glucose tolerance at baseline, despite the differing distributions of IFG and IGT.

This analysis demonstrates the utility of measuring circulating miRNAs with RNA sequencing from observational and interventional studies of type 2 diabetes to characterize changes that precede the development of diabetes. Results can be carried forward in the development of predictive biomarkers of future type 2 diabetes.

## Supplementary information

Supplementary Information.

## Data Availability

The datasets generated during and/or analysed during the current study are not publicly available due because this provision was not made in the original IRB application.
