# Supplementary material for: Short Report: Circulating microRNAs are associated with incident diabetes over 10 years in Japanese Americans
Source: Sci Rep. 2020 Apr 16;10:6509. doi: 10.1038/s41598-020-63606-3 (PMC7162904; doi:10.1038/s41598-020-63606-3)
Supplement: Supplementary file 1 — Supplementary Information. [file 41598_2020_63606_MOESM1_ESM.pdf]

Short Report: Circulating microRNAs are associated with incident diabetes over 10 years in Japanese Americans

Authors: Pandora L. Wander, Daniel A. Enquobahrie, Theo K. Bammler, Sengkeo Srinouanprachanh, James MacDonald, Steven E. Kahn, Donna Leonetti, Wilfred Y. Fujimoto, Edward J. Boyko

SUPPLEMENTAL ONLINE MATERIALS

Pre-processing, extraction, and profiling of circulating miRNAs

Lab personnel were blinded to individual participant outcomes. Because residual contamination of "cell free" samples such as plasma by blood cells, which are reservoirs of miRNA, is a limitation of previous circulating miRNA studies [1], thawed samples were spun at ~2500 RPMs for five minutes to completely clear the plasma of cells. Small RNAs were extracted from 500 μL aliquots of plasma using the Exiqon (now Qiagen) miRCURY™ RNA Biofluids Isolation Kit (Exiqon, Woburn, MA) [2]. We assessed the integrity, purity, and quantity of purified miRNA using spectrophotometry and an Agilent 2100 Bioanalyzer capillary electrophoresis system (Agilent Technologies Inc, Palo Alto, CA). Pico chip results showed a consistent peak at about 25 seconds, demonstrating that small RNAs were successfully extracted. Small chip results showed miRNA concentrations ranging from 38% to 61%. Concentrations ranged from 13.5 pg/μL to 85.4 pg/μL. RNA sequencing was performed by Qiagen, using unique molecular index barcodes to reduce bias introduced by PCR duplicates [3]. The Qiagen QIASeq MiRNA NGS Library Kit was used for library preparation. Plasma miRNAs were sequenced using an Illumina sequencer. We excluded miRNA transcripts based on the mean log counts/million counts (logCPM), with a mean logCPM <2.5 being exclusionary. Samples were normalized using a trimmed mean of M-values and counts converted to log counts/million (logCPM) using the voomWithQualityWeights function from the Bioconductor limma package.

Statistical analysis

We used number (%) and mean (standard deviation) for categorical and normally distributed continuous variables, respectively, to describe study population characteristics, both overall and stratified by sex. Analyses were conducted in R version 3.4.0. Because count data are not normally distributed and may have transcripts with zero counts [4], we used a linear model based on the negative binomial distribution in the Bioconductor edgeR package [5], making comparisons using quasi-likelihood F-tests [6]. An exploratory principal component analysis showed an apparent large batch effect captured by the first principal component, so we included the first principal component as a nuisance variable, along with two surrogate variables we detected using the Bioconductor sva package [7]. To protect against choosing miRNA transcripts that are statistically significantly differentially expressed, but at such a low fold change that differences are not biologically meaningful, we

made comparisons between individuals with and without incident diabetes, selecting miRNAs with a 25% difference [8] in expression as well as a <5% false discovery rate (FDR) [9].

Bioinformatics analysis

We used the Core Analysis feature of the Ingenuity Pathway Analysis (IPA) software program (Built version – 486617M; Content version – 33559992; Ingenuity Systems, A Qiagen Company, Redwood City, CA) to identify transcriptional networks using the 36 differentially expressed microRNAs [10]. We also used IPA's microRNA Target Filter to identify the mRNA targets of those 36 differentially expressed microRNAs. We restricted the microRNA targets to "experimentally validated" targets. We also performed IPA Core Analysis on the lists of microRNA targets that were identified as described above by IPA's microRNA Target Filter feature.

QIAseq miRNA Sequencing methods and QC

miRNA Sequencing Libraries. Five µl miRNA and 5 ul (100ng) total RNA were reverse transcribed into cDNA in 20 µl reactions using the QIAseq miRNA Library Kit (QIAGEN). miRNA libraries were generated in reaction with adapters containing Unique Molecular Index (UMI) ligated to the miRNA and amplified using PCR (19-22 cycles) and during which the PCR sequencing indices were added. After PCR the samples were purified. Libraries were quality assessed using TapeStation 4200 (Agilent).

Next-generation sequencing on Illumina NGS systems. miRNA sequencing libraries prepared with the QIAseq miRNA Library Kit were sequenced using Illumina NextSeq 500. Libraries were quantified, adjusted to 4nM each and pooled. The pooled libraries were further diluted down to 1.8 pM and loaded into a flow cell on NextSeq 500 according to the manufacturer instructions. The sequencing specifications were 1x76 bases and 12 million targeted reads per sample.

Reads Analysis. Raw reads were de-multiplexed and FASTQ files for each sample were generated using the bcl2fastq software (Illumina inc.). FASTQ data were checked using the FastQC tool. Reads and UMIs count were generated using GeneGlobe pipeline(QIAGEN). Breifly, during the QIAseq miRNA Library Kit construction process, each individual miRNA molecule was tagged with a Unique Molecular Index (UMI). Following sequencing and trimming, reads were analyzed for the presence of UMIs. All reads containing identical insert sequence and UMI sequence (insert-UMI pair) were collapsed into a single read. These reads

were passed into the analysis pipeline. Additionally reads containing partially UMI were also passed into the analysis pipeline. This allows for true quantification of the miRNAs by eliminating library amplification bias. Cutadapt (1.11) is used to extract information of adapter and UMI in raw reads, and output from Cutadapt is used to remove adapter sequences and to collapse reads by UMI.

REFERENCES

1 Pritchard, C. C. *et al.* Blood cell origin of circulating microRNAs: a cautionary note for cancer biomarker studies. *Cancer prevention research* **5**, 492-497, doi:10.1158/1940-6207.CAPR-11-0370 (2012).

2 Sedlackova, T., Repiska, G. & Minarik, G. Selection of an optimal method for co-isolation of circulating DNA and miRNA from the plasma of pregnant women. *Clinical chemistry and laboratory medicine : CCLM / FESCC* **52**, 1543-1548, doi:10.1515/cclm-2014-0021 (2014).

3 Fu, Y., Wu, P. H., Beane, T., Zamore, P. D. & Weng, Z. Elimination of PCR duplicates in RNA-seq and small RNA-seq using unique molecular identifiers. *BMC genomics* **19**, 531, doi:10.1186/s12864-018-4933-1 (2018).

4 Anders, S. *et al.* Count-based differential expression analysis of RNA sequencing data using R and Bioconductor. *Nat Protoc* **8**, 1765-1786, doi:10.1038/nprot.2013.099 (2013).

5 Robinson, M. D., McCarthy, D. J. & Smyth, G. K. edgeR: a Bioconductor package for differential expression analysis of digital gene expression data. *Bioinformatics* **26**, 139-140, doi:10.1093/bioinformatics/btp616 (2010).

6 Lund, S. P., Nettleton, D., McCarthy, D. J. & Smyth, G. K. Detecting differential expression in RNA-sequence data using quasi-likelihood with shrunken dispersion estimates. *Statistical applications in genetics and molecular biology* **11**, doi:10.1515/1544-6115.1826 (2012).

7 Leek, J. T., Johnson, W. E., Parker, H. S., Jaffe, A. E. & Storey, J. D. The sva package for removing batch effects and other unwanted variation in high-throughput experiments. *Bioinformatics* **28**, 882-883, doi:10.1093/bioinformatics/bts034 (2012).

8 McCarthy, D. J. & Smyth, G. K. Testing significance relative to a fold-change threshold is a TREAT. *Bioinformatics* **25**, 765-771, doi:10.1093/bioinformatics/btp053 (2009).

9 Benjamini, Y. & Hochberg, Y. Controlling the false discovery rate: a practical and powerful approach to multiple testing. *Journal of the Royal Statistical Society Series B* **57**, 289-300 (1995).

10 Kramer, A., Green, J., Pollard, J., Jr. & Tugendreich, S. Causal analysis approaches in Ingenuity Pathway Analysis. *Bioinformatics* **30**, 523-530, doi:10.1093/bioinformatics/btt703 (2014).

Supplementary Table 1. Pathways with activation z-score ≥ 2* in Ingenuity Pathway Analysis based on n=36 miRs that differed in plasma of individuals with and without incident diabetes at 10-year follow-up

Category	Function	Activation z-score	p-value
Cell Cycle, Embryonic Development	G1/S phase transition	2	4.41E-09
Cell Cycle, Connective Tissue Development and Function	G1/S phase transition	2	4.41E-08
Cell Cycle	interphase	2	4.46E-05
Cell Cycle	G1 phase	2	2.25E-04

* identifies potential regulators based on a statistically significant pattern match of up- or down-regulation

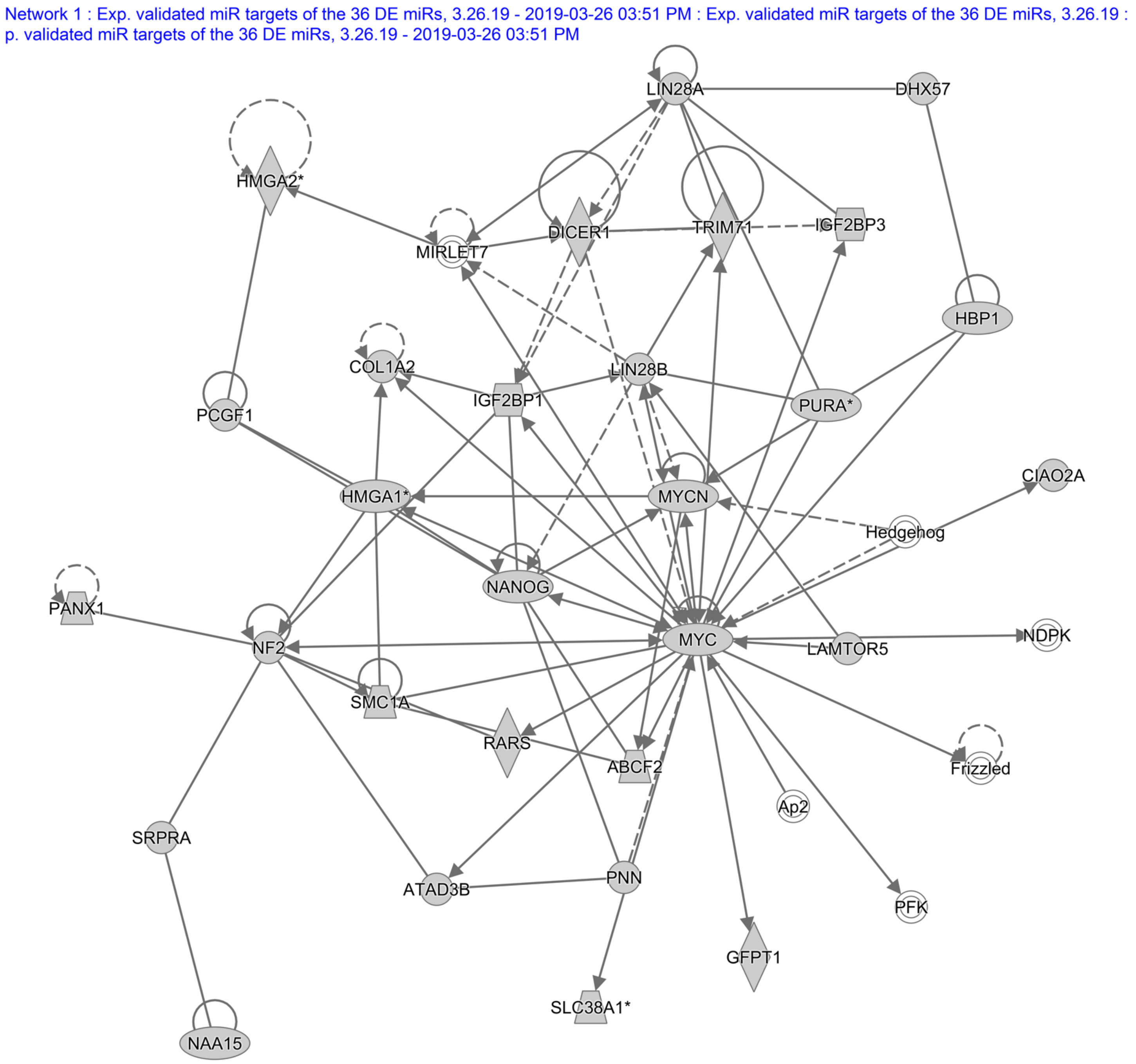

© 2000-2019 QIAGEN. All rights reserved.

Supplemental Fig. 1. Top network represented by experimentally validated gene (mRNA) targets of microRNAs that differed between individuals with and without incident diabetes at 10-year follow-up. The network was generated through the use of IPA (QIAGEN Inc., https://www.qiagenbio-informatics.com/products/ingenuity-pathway-analysis).
